# Nutlin-3a: A Potential Therapeutic Opportunity for *TP53* Wild-Type Ovarian Carcinomas

**DOI:** 10.1371/journal.pone.0135101

**Published:** 2015-08-06

**Authors:** Erin K. Crane, Suet-Yan Kwan, Daisy I. Izaguirre, Yvonne T. M. Tsang, Lisa K. Mullany, Zhifei Zu, JoAnne S. Richards, David M. Gershenson, Kwong-Kwok Wong

**Affiliations:** 1 Department of Gynecologic Oncology and Reproductive Medicine, The University of Texas MD Anderson Cancer Center, Houston, Texas, United States of America; 2 Department of Molecular and Cellular Biology, Baylor College of Medicine, Houston, Texas, United States of America; 3 Cancer Biology Program, The University of Texas at Houston Graduate School of Biomedical Sciences, Houston, Texas, United States of America; The University of Hong Kong, Queen Mary Hospital, HONG KONG

## Abstract

Epithelial ovarian cancer is a diverse molecular and clinical disease, yet standard treatment is the same for all subtypes. *TP53* mutations represent a node of divergence in epithelial ovarian cancer histologic subtypes and may represent a therapeutic opportunity in subtypes expressing wild type, including most low-grade ovarian serous carcinomas, ovarian clear cell carcinomas and ovarian endometrioid carcinomas, which represent approximately 25% of all epithelial ovarian cancer. We therefore sought to investigate Nutlin-3a—a therapeutic which inhibits MDM2, activates wild-type p53, and induces apoptosis—as a therapeutic compound for *TP53* wild-type ovarian carcinomas. Fifteen epithelial ovarian cancer cell lines of varying histologic subtypes were treated with Nutlin-3a with determination of IC_50_ values. Western Blot (WB) and quantitative real-time polymerase chain reaction (qRT-PCR) analyses quantified MDM2, p53, and p21 expression after Nutlin-3a treatment. DNA from 15 cell lines was then sequenced for *TP53* mutations in exons 2-11 including intron-exon boundaries. Responses to Nutlin-3a were dependent upon *TP53* mutation status. By qRT-PCR and WB, levels of MDM2 and p21 were upregulated in wild-type *TP53* sensitive cell lines, and p21 induction was reduced or absent in mutant cell lines. Annexin V assays demonstrated apoptosis in sensitive cell lines treated with Nutlin-3a. Thus, Nutlin-3a could be a potential therapeutic agent for ovarian carcinomas expressing wild-type *TP53* and warrants further investigation.

## Introduction

While ovarian cancer is the ninth most common cancer in the United States, it is the sixth most deadly: approximately 22,000 new cases of ovarian cancer are diagnosed annually, with 14,270 attributable deaths [1]. Epithelial ovarian cancer (EOC), which is thought to arise from the surface epithelium of the ovaries but could also be from extra-ovarian origins [2], accounts for over 90% of ovarian cancers. Within the epithelial category, several subtypes based on histopathogical criteria exist, including high-grade serous (70%), low-grade serous (<5%), clear cell (10%), endometrioid (10%), and mucinous (3%) [3]. Although high-grade serous ovarian carcinomas (SOCs) comprise the majority of epithelial ovarian cancer, less common subtypes account one third of all cases, many of which are chemoresistant and frequently with wild-type *TP53*. These histologic subtypes have distinct molecular origins, with correspondingly diverse and specific clinical behaviors; they are treated with the same regimen as high-grade SOC. For example, 96% of high-grade SOCs harbor *TP53* mutations [4] and rare high-grade SOC cases with wild-type *TP53* appear to be more chemo-resistant [5]. Clear cell ovarian carcinomas, on the other hand, typically express wild-type p53 but contain *ARIDIA* and *PI3K* aberrations, which often originate from endometriosis [6]; similarly, low-grade SOC also express wild-type p53 but contain *KRAS* or *BRAF* mutations and may be derived from serous borderline ovarian tumors [7, 8]. Clinically, low-grade SOCs affect younger patients and follow an indolent clinical course yet are relatively chemo-resistant, and patients eventually die of recurrent disease [9]. Whereas high-grade SOCs typically affect postmenopausal patients and are chemo-sensitive, median overall survival is only 54 months (compared to 126 months for low-grade) [10]. Those with advanced-stage clear cell carcinomas typically fare worse than those affected by high-grade SOC, partially due to their insensitivity to platinum-based chemotherapy [11].

Despite these discrepancies in molecular origins, mutational characteristics, chemo-sensitivity, and overall clinical behavior, the primary standard treatment remains the same for all histologic subtypes: platinum and taxane-based chemotherapy. The Gynecologic Oncology Group (GOG) has recently established a “Rare Tumor Committee” to develop and conduct definitive phase II trials for non-high grade serous ovarian cancer especially for low-grade serous and clear cell carcinomas. Innovative therapies are needed to improve the outcomes in these patient cohorts, and one obvious node of distinction between subtypes is the *TP53* pathway. Nutlin-3a is a small-molecule, murine double minute (MDM2) antagonist that inhibits MDM2-p53 interactions and stabilizes the p53 protein, thereby inducing cell cycle arrest and apoptosis [12]. We therefore sought to investigate Nutlin-3a as a potential therapeutic compound for *TP53* wild-type ovarian carcinomas.

## Materials and Methods

### Cell Lines

A total of 15 ovarian carcinoma cell lines were cultured: two low-grade serous (HOC7 and MPSC1); three clear cell (OVCA429, OVAS, TOV21G); five endometrioid (SKOV3, IGROV1, MDA2774, TOV112D, A2780); three mucinous (MCAS, RMUGL, RMUGS); and two high-grade serous (OVCAR-3, OVCA420). The SKOV3 cell line is an established *TP53*-mutant cell line which does not express *TP53* at the protein or mRNA level and was therefore used as a negative control [13]. HOC-7 [14] was a gift from Dr. Louis Dubeau at the University of Southern California, and MPSC1 [15] was a gift from Dr. Ie-Ming Shih at Johns Hopkins University. We have determined that cell line HOC-7 contains a *KRAS* mutation, cell line MPSC1 contains a *BRAF* mutation, and cell line TOV21G contains a *PIK3CA* mutation (data not shown). TOV21G, SKOV3, OVCAR-3 and TOV112D were obtained from ATCC (American Type Culture Collection). MDA2774 [16] was a gift from Dr. Ralph Freedman at MD Anderson. A2780 [17] was obtained from ECACC (European Collection of Cell Culture). MCAS, RMUGL and RMUGS were obtained from JCRB (Japanese Collection of Research Bioresources Cell Bank). IGROV1 was obtained from Dr. Susan Holbeck at National Cancer Institute [18]. OVCA420 and OVCA429 [19] were gifts from Dr. Robert Bast at MD Anderson Cancer Center. OVAS was a gift from Dr. Hiroaki Itamochi at Tottori University, Japan[20]. The MDM2 inhibitor Nutlin-3a was purchased from Selleck Chemicals (Houston, TX). Cell lines were incubated in a humidified atmosphere at 37°C with 5% CO_2_ and cultured in RPMI 1640 media supplemented with 10% fetal bovine serum, 100 U/ml penicillin, and 100 μg/ml streptomycin. For Western Blot and RT-PCR analyses, cell lines were treated with Nutlin-3a at their predetermined IC_50_, and protein and RNA were extracted at 24, 48, and 72 hours of treatment in conjunction with untreated controls. Media was exchanged with fresh Nutlin-3a every 24h.

### Determination of IC_50_ in Cell Lines

All 15 cell lines were plated at a density of 1 × 10^3^ cells per well in 96-well plates. After 24h, media was exchanged and cells were treated with incremental concentrations of Nutlin-3a (1 μM, 5 μM, 10 μM, 25 μM, 50 μM, and 70 μM). After 72h of incubation, WST-1 (Roche, Pleasanton, CA) was added to each well, and a microplate reader (BMG Labtech, Chicago, IL) was used at an absorbance of 450 nm to measure the number of remaining viable cells. Experiments were repeated with smaller titrations of Nutlin-3a as needed to determine the exact IC_50_ of cell lines. The IC_50_ was defined as the concentration at which a 50% reduction in cell viability occurred, which was calculated using Microsoft Excel 2010. Cell lines were again plated in a manner identical to above and treated with Nutlin-3a at their respective IC_50_, and WST-1 was added with cell viability measurement at 24, 48, and 72h.

### Sequencing for *TP53* mutations

DNA was extracted from cell lines according to manufacturer’s instructions using the Invitrogen Purelink Genomic DNA Mini Kit (Carlsbad, CA). DNA was amplified by polymerase chain reaction (PCR), and PCR products were then purified using the Invitrogen Purelink PCR Purification Kit. Exons 5–8 and exon 10 of *TP53* were then sequenced for mutation analysis in all samples via Sanger Sequencing at the MD Anderson Sequencing and Microarray Facility using the BigDye Terminator v3.1 Cycle Sequencing Kits and the 3730*xl* DNA Analyzer (Applied Biosystems, Carlsbad, CA). Sequences were then analyzed using both Finch TV v1.3.1 and Lasergene SeqMan Pro. The primer sequences for *TP53* sequencing are listed in the S1 Table.

### Western blot analysis

All 15 cell lines were examined for protein expression of p21 and p53 after treatment with Nutlin-3a at the IC_50_ dose via Western blot analysis. Protein lysates from cell cultures were extracted with radioimmunoprecipitation assay (RIPA) buffer and quantified by Bradford method. Electrophoresis of lysates (10 μg) was carried out on a 10% sodium dodecyl sulfate-polyacrylamide gel, followed by electroblot transfer onto a PVDF membrane. After blocking in 5% nonfat milk in phosphate-buffered saline (PBS) for 1 h, the membranes were probed with the following primary antibodies: Anti-mouse p21 (1:500 dilution; BD Pharmingen, San Diego, CA), anti-mouse p53 (1:1000 dilution; Santa Cruz Biotechnology, Inc., Santa Cruz, CA), and β-actin (1:50000 dilution; Sigma-Aldrich, St. Louis, MO) were dissolved in PBS with 5% bovine serum albumin (BSA), added to the Western blots, and incubated overnight at 4°C. The blots were then rinsed and incubated with IR-dye 680–conjugated secondary antibodies (LI-COR Biosciences, Lincoln, NE). Membranes were then imaged using the LI-COR Odyssey Infrared Image Detection System (LI-COR Biosciences, Lincoln, NE) at 700 nm and 800 nm.

### Quantitative real-time reverse transcriptase polymerase chain reaction

Gene expression of p53, p21 and MDM2 was determined by quantitative real-time reverse transcriptase polymerase chain reaction (qRT-PCR). After treatment at IC_50_ concentrations as described above, total RNA was extracted with the Ambion Minikit (Ambion, Carlsbad, CA) and cDNA was synthesized (High Capacity cDNA Archive kit, Applied Biosystems, Carlsbad, CA) according to manufacturer’s instructions. Using TaqMan primer sets for p53, p21 (cyclin-dependent kinase inhibitor 1), and MDM2, qRT-PCR was performed in triplicate with the housekeeping gene cyclophilin (PPIA; Applied Biosystems) as normalizer. The Bio-Rad C1000 Thermal Cycler (Bio-Rad Laboratories, Hercules, CA) was used for all reactions, and fold-change was calculated with the 2^-△△Ct^ method.

### APC Annexin V Staining

The two most sensitive cell lines (A2780, HOC7) were treated with Nutlin-3a at their respective IC_50_ and apoptosis was quantified at 24 hours of treatment with corresponding controls. Cells were centrifuged and the supernatant was removed. Annexin V APC (BD Pharmingen, San Diego, CA) was added to samples and incubated in the dark for 15 min, followed by washing with binding buffer and resuspension. The FACSAria cell sorter (BD Biosciences, San Jose, CA) was used to quantify Annexin V expression; data were analyzed with FlowJo v7.6.5.

### Statistical Analysis

SPSS 15.0 for Windows (SPSS, Inc.) was used to perform statistical analyses. A nonparametric Mann-Whitney test was used to assess the statistical significance of the differences in messenger RNA expression between RT-PCR samples. P values <0.05 were considered to be statistically significant.

### Identification of genes up-regulated in Nutlin-3a resistant cell lines with wild-type *TP53*

Gene expression data (Cel files) from 159 cancer cell lines with wild-type *TP53* and known Nutlin-3a sensitivity were downloaded from the Cancer Cell Line Encyclopedia study [21]. Cel files were processed with dChip software and normalized expressed data were used to identify differentially expressed genes by student t-test [22].

## Results

### Sensitivity to Nutlin-3a correlated with *TP53* mutation status

**Fig 1:**
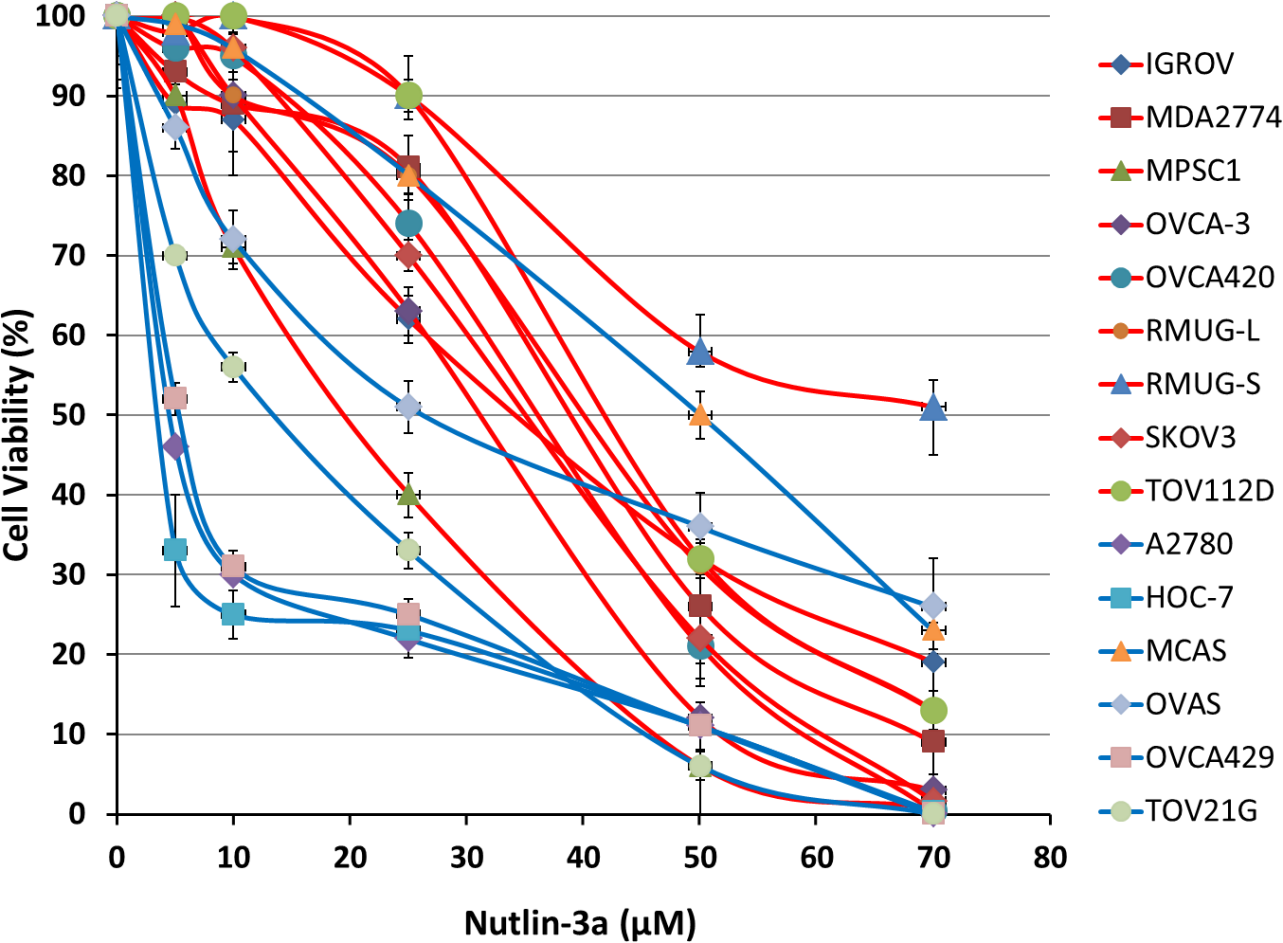
Cell viability of ovarian cancer cell lines treated with Nutlin-3a. All 15 cell lines were plated at a density of 1 × 10^3^ cells per well in 96-well plates. After 24h, media was exchanged and cells were treated with incremental concentrations of Nutlin-3a (1 μM, 5 μM, 10 μM, 25 μM, 50 μM, and 70 μM). After 72h of Nutlin-3a treatment, cell viability was measured by WST assay and compared to untreated control.

**Fig 2:**
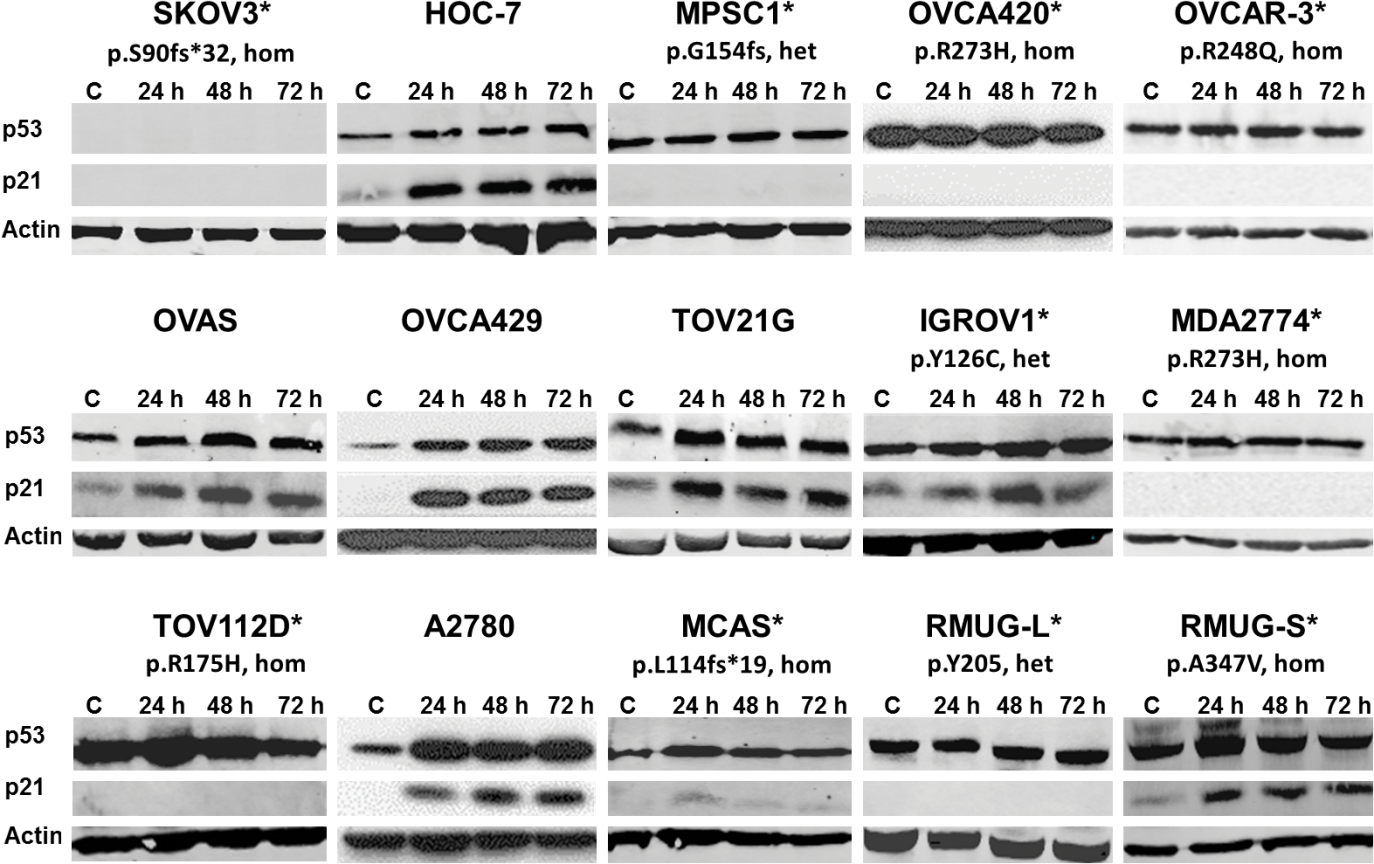
Protein expression of *TP53* and p21 of ovarian cancer cell lines after treated with Nutlin-3a for 24, 48 and 72 hours at their corresponding IC50 as indicated in Table 1. C, untreated control; *, cancer cell lines carrying *TP53* mutation. Het, heterozygous TP53 mutation; hom, homozygous *TP53* mutation.

The negative control cell line SKOV3 is known to contain a single nucleotide deletion in exon 4 [23] and was confirmed to have a single bp deletion in TP53 exon 4 (S1 Fig). The absence of the p53 protein and transcript expression in Western blot and qRT-PCR analyses confirmed the absence of p53 activity in SKOV3 (Figs 1 and 2). SKOV3 had an IC_50_ of 38 μM to Nutlin-3a. The DNA sequences of exon 2–11 of *TP53* in the tested cell lines and their sensitivity to Nutlin-3a were determined (Table 1, Fig 1). Nine cell lines (MPSC1, OVCA420, OVCAR-3, IGROV1, MDA2774, TOV112D, MCAS, RMUG-L and RMUG-S) were found to carry *TP53* mutations. The fluorescent sequencing chromatograms for the detected mutations for these nine cell lines are provided in the S1 Fig. All of these *TP53* mutant cell lines were quite resistant to Nutlin-3a (IC_50_ = 20 to >70 μM). Three cell lines (HOC-7, OVCA429 and A2780) with wild-type *TP53* were highly sensitive to Nutlin-3a (IC50 = 4 to 6 μM). HOC-7 is a low-grade SOC cell line with a known *KRAS* mutation [24]. However, the other low-grade SOC cell line MPSC1 was relatively more resistant (IC_50_ = 20 μM); correspondingly, this cell line was found to have a heterozygous *TP53* in-frame deletion (p.G154_S166delGTRVRAMAIYKQS) in exon 5 in this study. The Nutlin-3a sensitive OVCA429 cell line is an ovarian clear cell cell line, which has been shown to form clear cell adenocarcinoma when injected intraperitoneally in nude mice [25]. Furthermore, OVCA429 has a *PIK3CA* mutation, which is frequently activated in ovarian clear cell carcinomas [26]. The two remaining ovarian clear cell lines (TOV21G and OVAS), both with *TP53* wild-type, were relatively more sensitive to growth inhibition with Nutlin-3a (IC_50_ = 14 and 25 μm respectively) than the *TP53* mutant cell lines. The Nutlin-3a sensitive A2780 cell line is an endometrioid-like as should not be considered as a high grader serous ovarian cancer cell line as discussed recently. A2780 carries *PTEN*, *PIK3CA* and *ARID1A* mutations [27]. We also found that A2780 cell line had a heterozygous 16 bp insertion in the intron between *TP53* exon 3 and exon 4 without affecting the exons (S2 Fig). Moreover, no mutation was found in any of the other exons. All the other endometrioid cell lines carried *TP53* mutations and were Nutlin-3a resistant. The mucinous ovarian cancer cell lines appeared to be most resistant to Nutlin-3a (IC_50_ = 40 to > 70 μm). All these mucinous cell lines carried a *TP53* mutation. While RMUG-S and RMUG-L have missense mutations, MCAS cell line has a homozygous 127 bp deletion affecting the *TP53* exon 4 (S3 Fig).

**Table 1:** *TP53* mutation status and Nutlin-3a sensitivity of ovarian cancer cell lines.

Cell line	Histology	*TP53* status	Type	IC50 (Nutlin-3a) μM	Origin
HOC-7	Low-grade serous	wild-type		4	Buick et al (1985)[14]
MPSC1	Low-grade serous	c.460-499del40; p.G154_S166delGTRVRAMAIYKQS	Heterozygous	20	Pohl et al (2005)[15]
OVCA420	High-grade serous	c.818G>A; p.R273H	Homozygous	36	Bast et al (1981)[19]
OVCAR-3	High-grade serous	c.743 G>A; p.R248Q	Homozygous	31	ATCC
OVAS	Clear cell	wild-type		25	Morisawa et al (1988)[20]
OVCA429	Clear cell	wild-type		6	Bast et al (1981)[19]
TOV21G	Clear cell	wild-type		14	ATCC
IGROV1	Endometrioid	C.377 A>G; p.Y126C	Heterozygous	35	Benard et al (1985)[18]
MDA2774	Endometrioid	c.818G>A; p.R273H	Homozygous	40	Freedman et al (1978)[16]
TOV112D	Endometrioid	C.524 C>A; p.R175H	Homozygous	42	ATCC
A2780	Endometrioid	c.96+15_96+16insTCCAGGTCCCCAGCCC; wild-type	Heterozygous	5	Behrens et al (1987)[17]
MCAS	Mucinous	c.342-375del34+93; p.L114fs*19	Homozygous	50	JCRB
RMUG-L	Mucinous	c.614 A>G; Y205C	Heterozygous	40	JCRB
RMUG-S	Mucinous	c.1040 C>T; A347V	Homozygous	> 70	JCRB

ATCC, American Type Culture Collection; JCRB, Japanese Collection of Research Bioresources Cell Bank.

### Nutlin-3a induces upregulation of p53, p21 and MDM2

**Fig 3:**
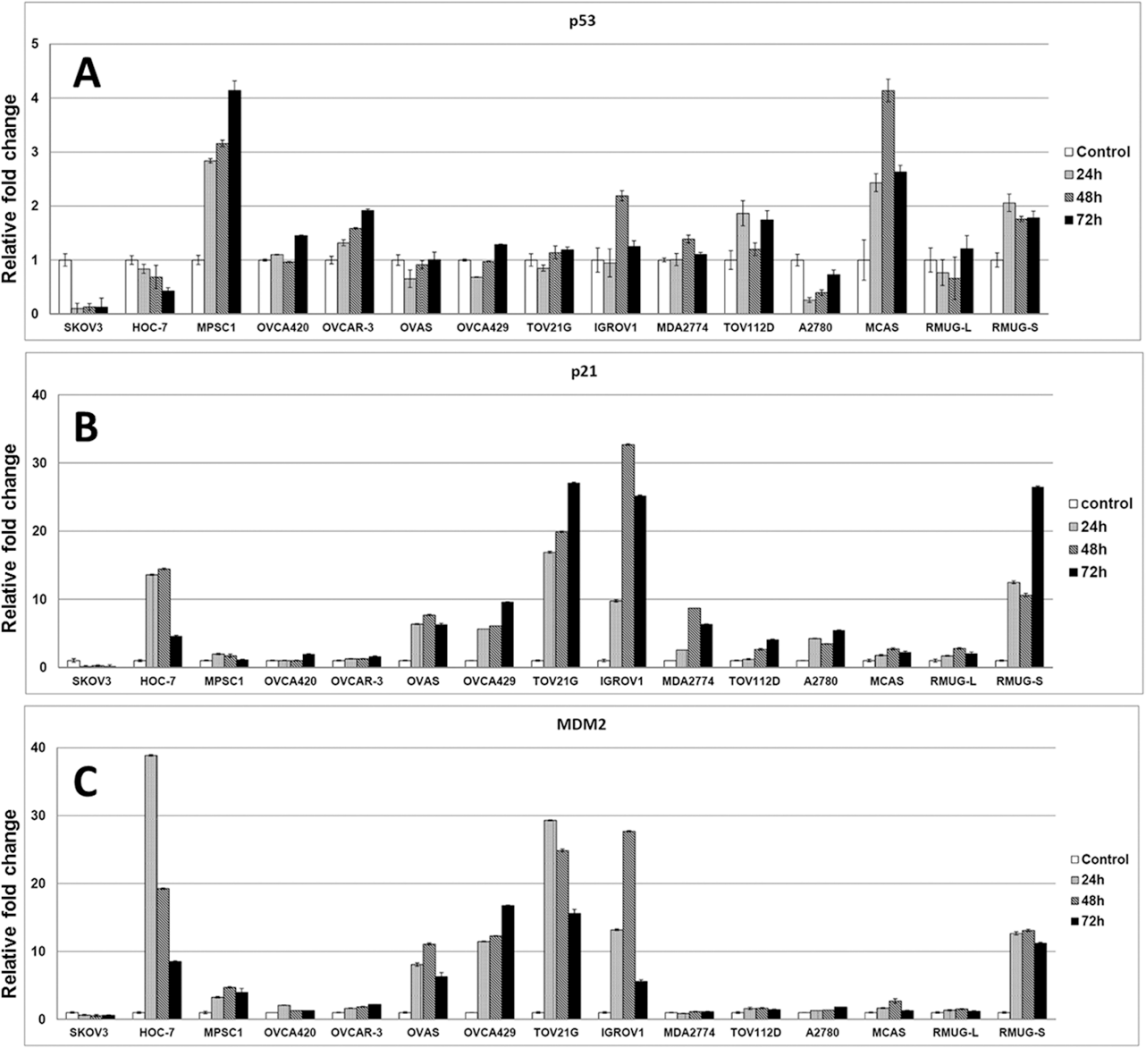
Gene expression of p21, *TP53*, and MDM2 of ovarian cancer cell lines after treated with Nutlin-3a for 24, 48 and 72 hours at their corresponding IC50 as indicated in Table 1. (A) *TP53*. (B) p21. (C) MDM2.

To examine the downstream effects of Nutlin-3a, Western blot and RT-PCR analyses were performed for p53, p21, and MDM2. The negative control cell line, SKOV3, exhibited no p53 protein and transcript (Figs 2 and 3). Cell lines with mutated *TP53* in general had higher expression of mutant forms of p53 protein. This is in agreement with the fact that mutant p53 proteins in tumor cells are stable because they are deficient in transactivating MDM2 [28]. For cell lines with wild-type *TP53*, an increase in the p53 protein expression and p21 protein expression was detected by Western blot (Fig 2). However, no increase in p53 protein expression or induction of p21 was detected in *TP53* mutant cell lines with the exception of IGROV1 and RMUG-S. A significant increase in the *TP53* transcripts was detected for the highly resistant MCAS cell line expressing a truncated *TP53* and the MPSC1 cell line with a wild-type *TP53* allele in the heterozygous *TP53* mutation background (Fig 3A). qRT-PCR analyses (Fig 3B) showed that the trend in p21 expression mirrored that of Western Blot. For sensitive cell lines, p21 was significantly up-regulated (p<0.05), with peak expression at 48–72 hours of exposure (Fig 3B). In general, cell lines with mutant p53 protein had lower level of MDM2 expression. Levels of MDM2 expression were concomitantly up-regulated with p21 in most wild-type cell lines (Fig 3C). Interestingly, RMUG-S—a mucinous cell line—displayed both p21 and MDM2 activity but expressed a *TP53* homozygous mutant and was the most resistant of all the cell lines.

### Nutlin-3a Induces Apoptosis

**Fig 4:**
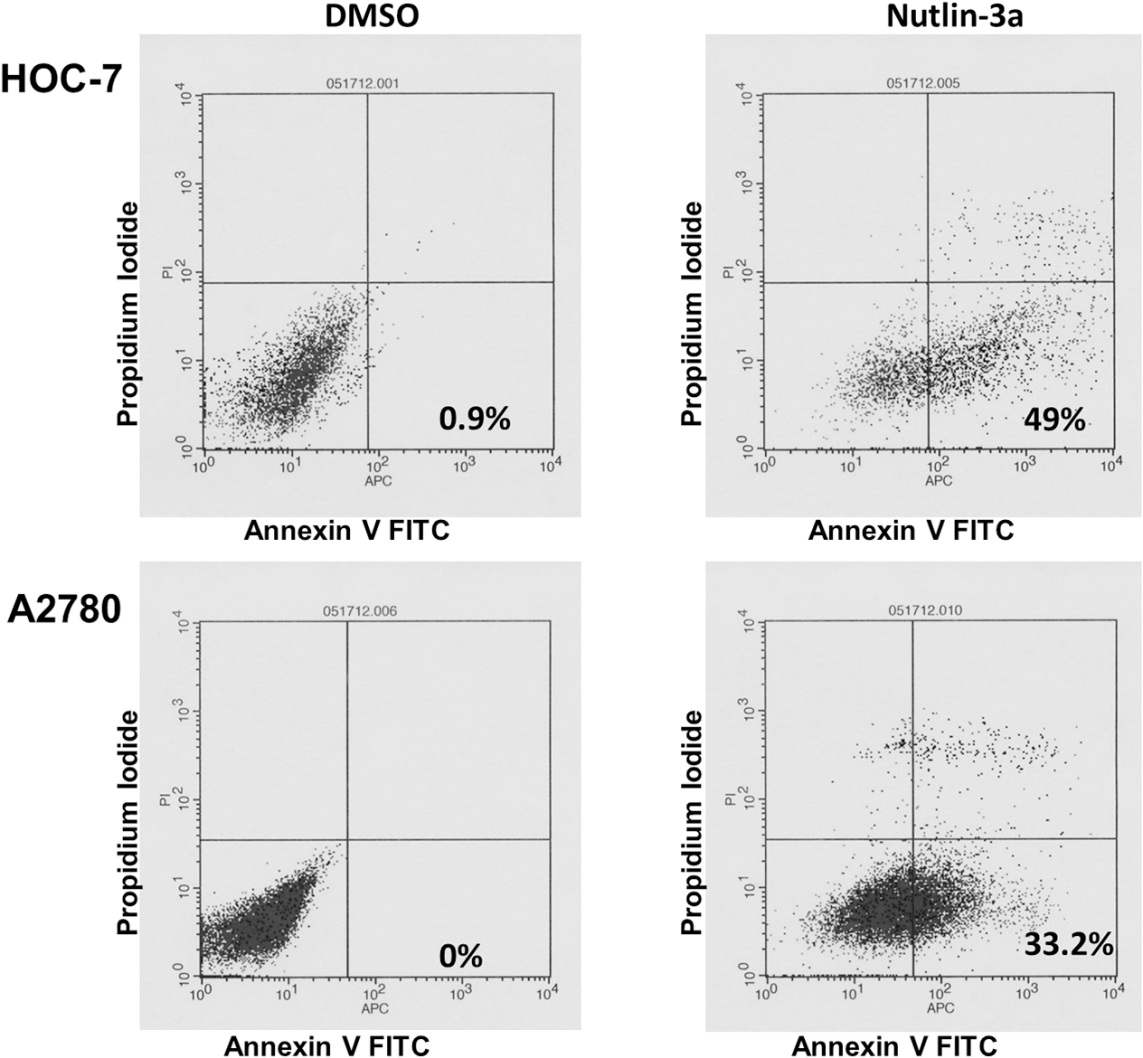
Cell death after Nutlin-3a exposure. Apoptosis was evaluated after treating HOC-7 and A2780 with 4 μM and 5 μM Nutlin-3a at their corresponding IC50 or DMSO control, and staining with Annexin-V at 24 h. The number represents the percentage of early apoptotic cells in each condition.

While Western blot, qRT-PCR, and cell proliferation assays demonstrated that Nutlin-3a induced cell cycle arrest via up-regulation of p21, we wished to determine whether it also affected apoptosis. Compared to untreated controls, flow cytometry of Annexin V stained cells demonstrated an induction of apoptosis in Nutlin-3a treated HOC-7 and A2780 cells with 49% and 33.2% of early apoptotic cells whichwere stained by Annexin V but not by propidium, respectively (Fig 4).

### Identification of up-regulated genes in Nutlin-3a resistant wild-type cell lines

To identify genes that might be responsible for Nutlin-3a resistance, comparison of the gene expression profiles between 23 Nutlin-3a sensitive cell lines and 136 Nutlin-3a resistant cell lines were performed. The list of cell lines with wild-type *TP53* and known Nutlin-3a sensitivity from the CCLE project is provided in S2 Table. The list of 123 significantly up-regulated genes in Nutlin-3a resistant cell lines is listed in S3 Table. Since only one Nutlin-3a sensitive ovarian cancer cell line (A2780) with wild-type *TP53* was available in the CCLE study, additional gene expression profiles for two Nutlin-3a sensitive cell lines (HOC-7 and OVCA429) identified in this study were generated to further delineate up-regulated genes that might be relevant to ovarian cancer cells. The gene expression profiles of three Nutlin-3a sensitive ovarian cancer cell lines (A2780, HOC-7 and OVCA429) were compared to six Nutlin-3a resistant ovarian cancer cell lines (HeyA8, EFO21, MCAS, OC316, OVTOKO and TOV21G). The 208 significantly up-regulated genes in Nultin-3a resistant ovarian cancer cell lines are listed in S4 Table. Four genes (Table 2) were found to be shared between the two lists (S3 Table and S4 Table). One of the genes (GDA) may be related to apoptosis and the other three genes (CXLC5, CCL20 and MAP7) are related to cell proliferation.

**Table 2:** List of genes significantly up-regulated in Nutlin-3a resistant cancer cell lines with wild-type *TP53*.

probe set	gene	Nutlin-3a sensitive cells (n = 23) mean expression value	Nutlin-3a resistant cells (n = 136) mean expression value	fold change	P value
224209_s_at	GDA: guanine deaminase	37.7	703.1	18.6	0.000003
207852_at	CXCL5: chemokine (C-X-C motif) ligand 5	14.3	244.6	17.1	0.001889
205476_at	CCL20: chemokine (C-C motif) ligand 20	97.0	386.2	4.0	0.003113
202890_at	MAP7: microtubule-associated protein 7	127.8	491.7	3.9	0.00002

## Discussion

Deemed “the guardian of the genome,” the tumor protein 53 gene *TP53* harbors a set of diverse and complex functions which protect the cell from genomic damage and ensure genomic integrity. The “protective” functions appear to occur with low levels of wild-type p53; only at higher levels of activity does p53 act to terminate cell proliferation and induce apoptosis [29]. Naturally, restoration or enhancement of elevated wild-type *TP53* activity is an attractive anti-cancer strategy, as *TP53* is altered in ~50% of human cancers [30]. *TP53* mutations are virtually ubiquitous in high-grade serous ovarian carcinomas; however, this is not the case for other EOCs.

Nutlin-3a belongs to a class of compounds initially described by Vassilev *et al* [12] and functions by inhibition of MDM2-p53 binding, and thereby prevents p53 ubiquitination by MDM2 leading to p53 stabilization and increased wild-type *TP53* activity. Others have validated the activity of Nutlin-3a in neuroblastoma, T-cell lymphoma, gastrointestinal stromal tumors [31], sarcomas [32], renal cell carcinomas [33], and colorectal carcinomas [34], among others [35]. While the compound has been tested in other tumor types, its utility in ovarian cancer has been largely overlooked, as most clinical efforts are directed towards the most common histologic subtype—high-grade SOC—which is *TP53* mutant. In our study, we demonstrate that Nutlin-3a has activity in *TP53* wild-type ovarian carcinomas, requires an intact p53 pathway for efficacy, increases p21, and results in apoptosis. We have previously documented the utility of Nutlin-3a in low-grade serous ovarian carcinoma, with up-regulation of cell cycle control, and apoptosis genes including *CDKN1A*, *CDKN2A*, *PERP*, and *PUMA* [29]. Here, we expand the efficacy of Nutlin-3a to other *TP53* wild-type epithelial ovarian carcinomas which includes a low-grade SOC cell line HOC-7, an endometrioid-like cell line A2780 and a clear cell cell line OVCA429 and demonstrate that it directly enhances apoptosis.

Two clear cell cell line (OVAS and TOV21G) with wild-type *TP53* were also relative sensitive to Nutlin-3a. On the other hand, all the mucinous cell lines had *TP53* mutation and were extremely resistant to Nutlin-3a. Advanced-stage mucinous ovarian carcinomas portend a poorer prognosis than their epithelial counterparts and are chemoresistant [36], and controversy exists as to whether these tumors are truly of ovarian versus gastrointestinal origin. As several factors influence the cellular response to targeted therapy, it is possible that these cell lines contain additional mutational or epigenetic properties that render them resistant to Nutlin-3a. One limitation to this study is the lack of an *in vivo* model; these investigations are currently ongoing.

To further investigate the possible mechanism of resistance, we compared the gene expression profiles from 23 Nutlin-3a resistant cell lines and 136 Nutlin-3a resistant cell lines using data from the CCLE study. Four genes were found to be highly up-regulated in Nutlin-3a resistant cell lines with wild-type *TP53* (Table 2). One of these genes may be involved in apoptosis, and three other genes are involved in inflammation or cell proliferation. Guanine deaminase is an enzyme that converts guanine to xanthine and ammonia, which can generate reactive oxygen species (ROS) [37]. ROS plays an important role in the process of apoptosis in many cell types [38]. MAP7 is a microtubule-associated protein. Previous studies have shown that transfecting the human lung adenocarcinoma cell line A549 with a MAP7 overexpressing plasmid significantly increases the cell proliferation [39]. CXCL5 and CCL20 are both chemokines. CXCL5 is required for cell proliferation of head and neck squamous cell carcinoma [40]. More interestingly, gain of function of *TP53* mutation has been shown to up-regulate CXCL5 [41]. Whether these Nutlin-3a resistant cell lines might have any other gain of function mutations will need further investigation. The chemokine CCL20 has been reported to promote cancer cell proliferation and migration through the chemokine receptor CCR6 [42]. These genes could be potential biomarkers for predicting Nutlin-3a resistance.

While inherent resistance to Nutlin-3a exists in *TP53* mutant carcinomas, acquired resistance to Nutlin-3 may occur via acquisition of *de novo TP53* mutations [43] or overexpression of MDM4 [44]. Others have shown that p21 induction does not necessarily affect the apoptotic response to nongenotoxic *TP53* activation by nultin-3a [45]. As shown in our study, Nutlin-3a highly induced p21 protein expression in two Nutlin-3a resistant IGROV1 and RMUG-S cell lines. IGROV1 had a heterozygous *TP53* mutation (p.Y126C) in the DNA binding domain. It is possible that Y126C is not a dominant negative mutant and the wild-type p53 is still functional to induce p21 protein expression in the presence of Nutlin-3a. On the other hand, RMUG-S had a homozygous *TP53* mutation (A347V) in the tetramerisation motif and the mutant protein might still have DNA binding activity that could activate p21 protein expression. Logically, it follows that addition of cytotoxic agents may improve chemo-sensitivity—this has been demonstrated in colon, breast, and hepatocellular carcinoma cell lines, as well as melanoma and sarcoma [46].

In the clinical arena, at least six phase I trials employing Nutlin-3a have been recently completed in hematologic malignancies, solid tumors, and in combination with doxorubicin in sarcomas (NCT00559533, NCT00623870, NCT01143740, NCT01164033, NCT01635296, NCT01605526). Preliminary clinical data indicate that RG7112 (an oral formulation of nutlin-3a) appears to be well-tolerated in patients and indicates initial evidence of clinical activity [47, 48]. Given the poor prognosis of epithelial ovarian cancer with wild-type *TP53* (25–30% of all EOCs) and a relative lack of success with targeted agents in this field, we assert that further clinical investigation into the utility of Nutlin-3a in *TP53* wild-type epithelial ovarian carcinomas is warranted.

## Supporting Information

S1 Fig Fluorescent peak trace chromatograms showing *TP53* mutations in nine ovarian cancer cell lines. (TIF)

S2 Fig Fluorescent peak trace chromatograms showing a 16 bp heterozygous insertion in the intron between *TP53* exon 3 and exon 4 of A2780 cell line. (TIF)

S3 Fig Fluorescent peak trace chromatograms showing a 127 bp homozygous deletion in the *TP53* exon 4 of MCAS cell line. (TIF)

S1 Table List of primers for PCR amplication and sequencing of exon 2 to exon 11 of *TP53*. (DOCX)

S2 Table List of cancer cell lines form Cancer Cell Line Encyclopdia (CCLE) with wild-type *TP53* and known sensitivity to Nutlin-3a. (DOCX)

S3 Table List of up-regulated genes in all CCLE Nutlin-3a resistant cell lines in comparison to all CCLE sensitive cell lines. (XLSX)

S4 Table List of up-regulated genes in Nutlin-3a resistant ovarian cancer cell lines in comparison to all sensitive ovarian cancer cell lines with wild-type *TP53*. (XLSX)
