# Supplementary figures and images for: Nutlin-3a: A Potential Therapeutic Opportunity for TP53 Wild-Type Ovarian Carcinomas
Source: PLoS One. 2015 Aug 6;10(8):e0135101. doi: 10.1371/journal.pone.0135101 (PMC4527847; doi:10.1371/journal.pone.0135101)

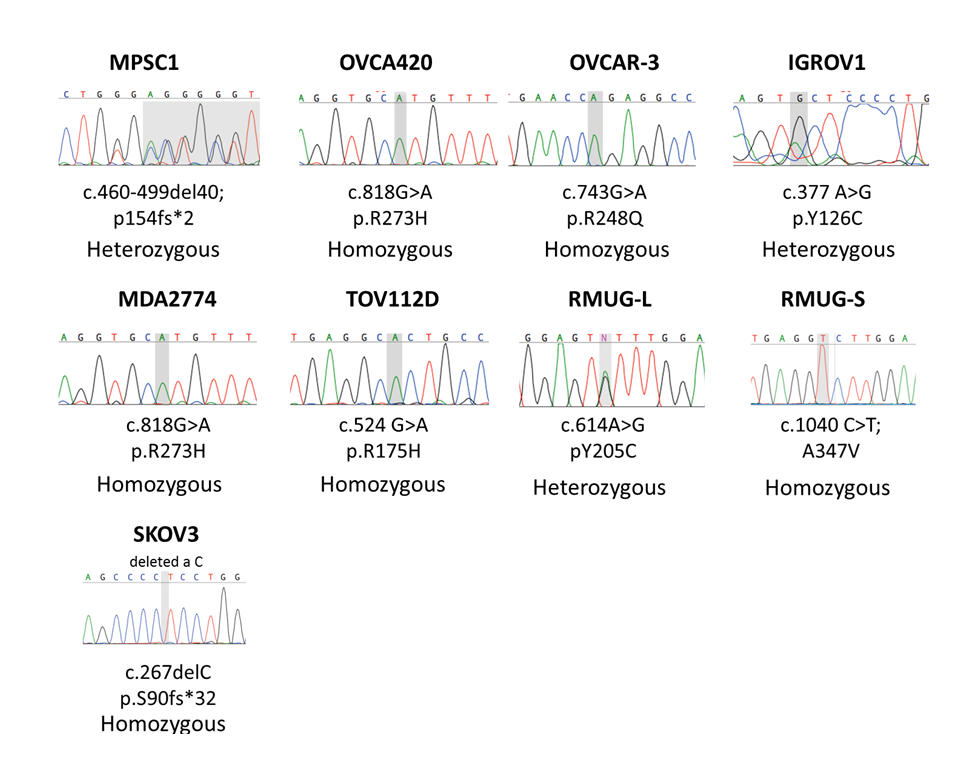

Supplement: S1 Fig — (TIF) [file pone.0135101.s001.tif]

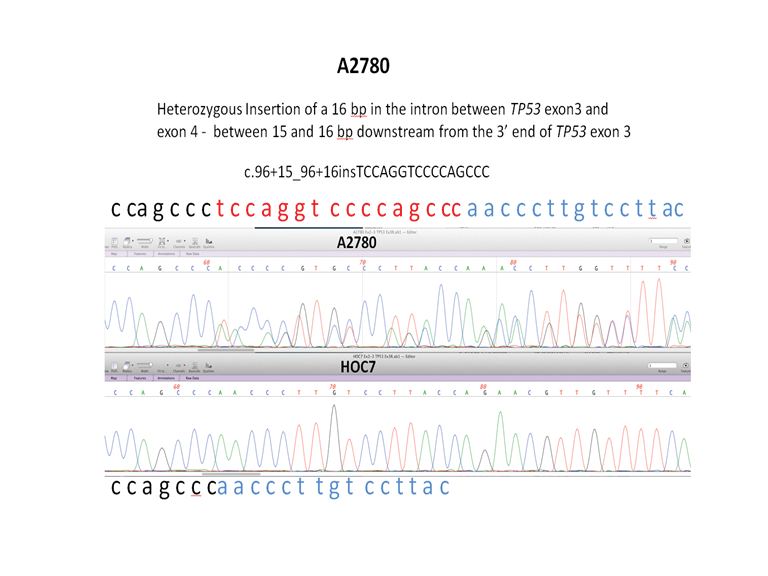

Supplement: S2 Fig — (TIF) [file pone.0135101.s002.tif]

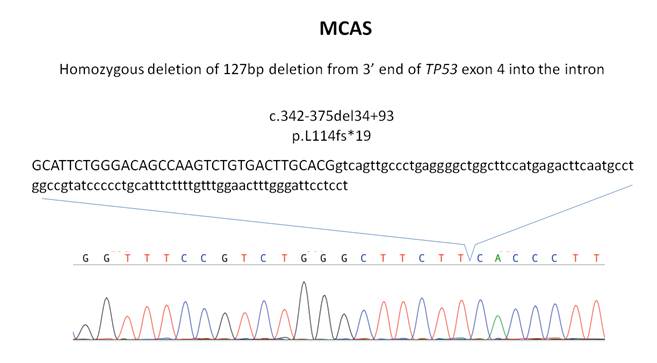

Supplement: S3 Fig — (TIF) [file pone.0135101.s003.tif]
