# Supplementary material for: Nutlin-3a: A Potential Therapeutic Opportunity for TP53 Wild-Type Ovarian Carcinomas
Source: PLoS One. 2015 Aug 6;10(8):e0135101. doi: 10.1371/journal.pone.0135101 (PMC4527847; doi:10.1371/journal.pone.0135101)
Supplement: S1 Table — (DOCX) [file pone.0135101.s004.docx]

| **S1_Table. List of primers for PCR amplication and sequencing of exon2 to exon 11 of *TP53*.** | |
| --- | --- |
|  |  |
| p53 Exon2 F | AGGGTTGGAAGTGTCTCATGC |
| p53 Exon3 R | AAATCATCCATTGCTTGGGAC |
| p53 Exon4 F | ACGTTCTGGTAAGGACAAGGG |
| p53 Exon4 R | GAGGAATCCCAAAGTTCCAAAC |
| p53 Exon4 R2 | GTCAGAGATCACACATTAAGTGGG |
| p53 Exon5-6 F | TGTTCACTTGTGCCCTGACT |
| p53 Exon5-6 R | GAGGGCCACTGACAACCA |
| p53 Exon7 F | AGGTCTCCCCAAGGCGCACTG |
| p53 Exon7 R | TGTGCAGGGTGGCAAGTGGC |
| p53 Exon8 F | TGGGAGTAGATGGAGCCTGG |
| p53 Exon8 R | AGGAAAGAGGCAAGGAAAGG |
| p53 Exon9 R | TTGTCTTTGAGGCATCACTGC |
| p53 Exon10 F | ATTGCACCATTGCACTCCC |
| p53 Exon10 R | AGCTGCCTTTGACCATGAAG |
| p53 Exon11 F | CCATCTTGATTTGAATTCCCG |
| p53 Exon11 R | ATTGCAAGCAAGGGTTCAAAG |
