# Supplementary material for: Nutlin-3a: A Potential Therapeutic Opportunity for TP53 Wild-Type Ovarian Carcinomas
Source: PLoS One. 2015 Aug 6;10(8):e0135101. doi: 10.1371/journal.pone.0135101 (PMC4527847; doi:10.1371/journal.pone.0135101)
Supplement: S2 Table — (DOCX) [file pone.0135101.s005.docx]

| **S2_Table. List of cancer cell lines form Cancer Cell Line Encyclopdia (CCLE) with wild-type p53 and known sensitivity to Nutlin-3a** | | |  |
| --- | --- | --- | --- |
|  |  |  |  |
| **Array** | **Cell line_Primary tissue** | **Nutlin IC50 uM** | **Nutlin-3 sensitivity** |
| NIECE_p_NCLE_RNA3_HG-U133_Plus_2_C09_296054 | TE11_OESOPHAGUS | 0.01 | Sensitive |
| WATCH_p_NCLE_RNA8_HG-U133_Plus_2_C10_474758 | JVM3_HAEMATOPOIETIC_AND_LYMPHOID_TISSUE | 0.27 | Sensitive |
| WATCH_p_NCLE_RNA8_HG-U133_Plus_2_G01_474646 | SIGM5_HAEMATOPOIETIC_AND_LYMPHOID_TISSUE | 1.45 | Sensitive |
| SILOS_p_NCLE_RNA9_HG-U133_Plus_2_F09_523480 | DOHH2_HAEMATOPOIETIC_AND_LYMPHOID_TISSUE | 2.60 | Sensitive |
| MAKER_p_NCLE_RNA7_HG-U133_Plus_2_C04_454614 | ZR751_BREAST | 2.73 | Sensitive |
| GILDS_p_NCLE_RNA11_Redo_HG-U133_Plus_2_G02_587654 | IMR32_AUTONOMIC_GANGLIA | 2.92 | Sensitive |
| AGENT_p_NCLE_RNA6_HG-U133_Plus_2_F02_436470 | MSTO211H_PLEURA | 3.37 | Sensitive |
| SILOS_p_NCLE_RNA9_HG-U133_Plus_2_G10_523540 | OCIAML5_HAEMATOPOIETIC_AND_LYMPHOID_TISSUE | 3.42 | Sensitive |
| MAKER_p_NCLE_RNA7_HG-U133_Plus_2_C09_454626 | SNU1_STOMACH | 3.71 | Sensitive |
| BUNDS_p_NCLE_RNA5_HG-U133_Plus_2_G11_419824 | KE97_HAEMATOPOIETIC_AND_LYMPHOID_TISSUE | 3.84 | Sensitive |
| METIS_p_NCLE_RNA1_Human_U133_Plus_2.0_C02_240900 | A2780_OVARY | 4.07 | Sensitive |
| BUNDS_p_NCLE_RNA5_HG-U133_Plus_2_B11_419860 | KPNSI9S_AUTONOMIC_GANGLIA | 4.26 | Sensitive |
| WATCH_p_NCLE_RNA8_HG-U133_Plus_2_H12_474618 | MOLP8_HAEMATOPOIETIC_AND_LYMPHOID_TISSUE | 4.62 | Sensitive |
| CASED_p_NCLE_RNA4_HG-U133_Plus_2_E04_383676 | G401_SOFT_TISSUE | 5.31 | Sensitive |
| WATCH_p_NCLE_RNA8_HG-U133_Plus_2_H11_474690 | AMO1_HAEMATOPOIETIC_AND_LYMPHOID_TISSUE | 5.46 | Sensitive |
| METIS_p_NCLE_RNA1_Human_U133_Plus_2.0_A05_240858 | 769P_KIDNEY | 5.59 | Sensitive |
| AGENT_p_NCLE_RNA6_HG-U133_Plus_2_D08_436576 | HT1080_SOFT_TISSUE | 6.07 | Sensitive |
| BRAKE_p_NCLE_RNA2_HG-U133_Plus_2_C09_241106 | SJSA1_BONE | 6.46 | Sensitive |
| SILOS_p_NCLE_RNA9_HG-U133_Plus_2_G07_523548 | OCIAML2_HAEMATOPOIETIC_AND_LYMPHOID_TISSUE | 6.55 | Sensitive |
| METIS_p_NCLE_RNA1_Human_U133_Plus_2.0_H06_241028 | COLO679_SKIN | 6.98 | Sensitive |
| MAKER_p_NCLE_RNA7_HG-U133_Plus_2_G12_454736 | REH_HAEMATOPOIETIC_AND_LYMPHOID_TISSUE | 7.59 | Sensitive |
| BUNDS_p_NCLE_RNA5_HG-U133_Plus_2_F07_419790 | SKNSH_AUTONOMIC_GANGLIA | 7.61 | Sensitive |
| NIECE_p_NCLE_RNA3_HG-U133_Plus_2_D05_296070 | JHUEM2_ENDOMETRIUM | 7.96 | Sensitive |
| METIS_p_NCLE_RNA1_Human_U133_Plus_2.0_H02_241020 | A172_CENTRAL_NERVOUS_SYSTEM | >8 | resistant |
| BRAKE_p_NCLE_RNA2_HG-U133_Plus_2_G04_241192 | A204_SOFT_TISSUE | >8 | resistant |
| METIS_p_NCLE_RNA1_Human_U133_Plus_2.0_H01_241018 | A549_LUNG | >8 | resistant |
| CASED_p_NCLE_RNA4_HG-U133_Plus_2_B01_383784 | A673_BONE | >8 | resistant |
| NIECE_p_NCLE_RNA3_HG-U133_Plus_2_E08_296100 | ACHN_KIDNEY | >8 | resistant |
| SILOS_p_NCLE_RNA9_HG-U133_Plus_2_C02_523526 | ALLSIL_HAEMATOPOIETIC_AND_LYMPHOID_TISSUE | >8 | resistant |
| NIECE_p_NCLE_RNA3_HG-U133_Plus_2_H04_296164 | BDCM_HAEMATOPOIETIC_AND_LYMPHOID_TISSUE | >8 | resistant |
| PARES_p_NCLE_RNA_Redo_HG-U133_Plus_2_A09_257614 | BT549_BREAST | >8 | resistant |
| NIECE_p_NCLE_RNA3_HG-U133_Plus_2_F07_296122 | C32_SKIN | >8 | resistant |
| AGENT_p_NCLE_RNA6_HG-U133_Plus_2_E07_436554 | C3A_LIVER | >8 | resistant |
| PARES_p_NCLE_RNA_Redo_HG-U133_Plus_2_C04_257652 | CAKI2_KIDNEY | >8 | resistant |
| PARES_p_NCLE_RNA_Redo_HG-U133_Plus_2_D07_257682 | CALU1_LUNG | >8 | resistant |
| METIS_p_NCLE_RNA1_Human_U133_Plus_2.0_B09_240890 | CAPAN2_PANCREAS | >8 | resistant |
| NIECE_p_NCLE_RNA3_HG-U133_Plus_2_G10_296152 | CHP212_AUTONOMIC_GANGLIA | >8 | resistant |
| CASED_p_NCLE_RNA4_HG-U133_Plus_2_B03_383776 | COLO201_LARGE_INTESTINE | >8 | resistant |
| AGENT_p_NCLE_RNA6_HG-U133_Plus_2_B04_436594 | COLO205_LARGE_INTESTINE | >8 | resistant |
| CASED_p_NCLE_RNA4_HG-U133_Plus_2_A01_383726 | CORL23_LUNG | >8 | resistant |
| METIS_p_NCLE_RNA1_Human_U133_Plus_2.0_F10_240988 | DBTRG05MG_CENTRAL_NERVOUS_SYSTEM | >8 | resistant |
| CRAZY_p_NCLE_RNA10_HG-U133_Plus_2_H11_569412 | DKMG_CENTRAL_NERVOUS_SYSTEM | >8 | resistant |
| MAKER_p_NCLE_RNA7_HG-U133_Plus_2_C03_454616 | DV90_LUNG | >8 | resistant |
| PARES_p_NCLE_RNA_Redo_HG-U133_Plus_2_A12_257620 | EFO21_OVARY | >8 | resistant |
| NIECE_p_NCLE_RNA3_HG-U133_Plus_2_B09_296030 | EN_ENDOMETRIUM | >8 | resistant |
| NIECE_p_NCLE_RNA3_HG-U133_Plus_2_A08_296004 | G361_SKIN | >8 | resistant |
| NIECE_p_NCLE_RNA3_HG-U133_Plus_2_G06_296144 | G402_SOFT_TISSUE | >8 | resistant |
| SILOS_p_NCLE_RNA9_HG-U133_Plus_2_A06_523556 | GRANTA519_HAEMATOPOIETIC_AND_LYMPHOID_TISSUE | >8 | resistant |
| CASED_p_NCLE_RNA4_HG-U133_Plus_2_D12_383660 | H4_CENTRAL_NERVOUS_SYSTEM | >8 | resistant |
| CASED_p_NCLE_RNA4_HG-U133_Plus_2_G09_383594 | HCC1187_BREAST | >8 | resistant |
| SILOS_p_NCLE_RNA9_HG-U133_Plus_2_A05_523512 | HCC1806_BREAST | >8 | resistant |
| WATCH_p_NCLE_RNA8_HG-U133_Plus_2_A11_474652 | HCC4006_LUNG | >8 | resistant |
| METIS_p_NCLE_RNA1_Human_U133_Plus_2.0_G05_241002 | HCT116_LARGE_INTESTINE | >8 | resistant |
| AGENT_p_NCLE_RNA6_HG-U133_Plus_2_F01_436468 | HEC151_ENDOMETRIUM | >8 | resistant |
| BUNDS_p_NCLE_RNA5_HG-U133_Plus_2_F03_419766 | HEC265_ENDOMETRIUM | >8 | resistant |
| GILDS_p_NCLE_RNA11_Redo_HG-U133_Plus_2_H01_587734 | HEC59_ENDOMETRIUM | >8 | resistant |
| BUNDS_p_NCLE_RNA5_HG-U133_Plus_2_F10_419784 | HEC6_ENDOMETRIUM | >8 | resistant |
| METIS_p_NCLE_RNA1_Human_U133_Plus_2.0_A09_240866 | HEPG2_LIVER | >8 | resistant |
| METIS_p_NCLE_RNA1_Human_U133_Plus_2.0_G01_240994 | HEYA8_OVARY | >8 | resistant |
| MAKER_p_NCLE_RNA7_HG-U133_Plus_2_E03_454660 | HS229T_LUNG | >8 | resistant |
| METIS_p_NCLE_RNA1_Human_U133_Plus_2.0_D06_240932 | HS695T_SKIN | >8 | resistant |
| METIS_p_NCLE_RNA1_Human_U133_Plus_2.0_B12_240896 | HS766T_PANCREAS | >8 | resistant |
| MAKER_p_NCLE_RNA7_HG-U133_Plus_2_D07_454646 | HS840T_UPPER_AERODIGESTIVE_TRACT | >8 | resistant |
| WATCH_p_NCLE_RNA8_HG-U133_Plus_2_A06_474676 | HS852T_SKIN | >8 | resistant |
| AGENT_p_NCLE_RNA6_HG-U133_Plus_2_B12_436534 | HS895T_SKIN | >8 | resistant |
| AGENT_p_NCLE_RNA6_HG-U133_Plus_2_H03_436486 | HS936T_SKIN | >8 | resistant |
| PARES_p_NCLE_RNA_Redo_HG-U133_Plus_2_B07_257634 | HS944T_SKIN | >8 | resistant |
| MAKER_p_NCLE_RNA7_HG-U133_Plus_2_B08_454598 | HT144_SKIN | >8 | resistant |
| PARES_p_NCLE_RNA_Redo_HG-U133_Plus_2_B02_257624 | HT29_LARGE_INTESTINE | >8 | resistant |
| PARES_p_NCLE_RNA_Redo_HG-U133_Plus_2_C09_257662 | IM95_STOMACH | >8 | resistant |
| AGENT_p_NCLE_RNA6_HG-U133_Plus_2_G09_436490 | ISTMES1_PLEURA | >8 | resistant |
| PARES_p_NCLE_RNA_Redo_HG-U133_Plus_2_C08_257660 | JHH2_LIVER | >8 | resistant |
| CRAZY_p_NCLE_RNA10_HG-U133_Plus_2_E01_569420 | JHH6_LIVER | >8 | resistant |
| CASED_p_NCLE_RNA4_HG-U133_Plus_2_H02_383622 | JM1_HAEMATOPOIETIC_AND_LYMPHOID_TISSUE | >8 | resistant |
| METIS_p_NCLE_RNA1_Human_U133_Plus_2.0_C12_240920 | K029AX_SKIN | >8 | resistant |
| SILOS_p_NCLE_RNA9_HG-U133_Plus_2_H02_523610 | KARPAS422_HAEMATOPOIETIC_AND_LYMPHOID_TISSUE | >8 | resistant |
| BUNDS_p_NCLE_RNA5_HG-U133_Plus_2_C08_419728 | KHM1B_HAEMATOPOIETIC_AND_LYMPHOID_TISSUE | >8 | resistant |
| PARES_p_NCLE_RNA_Redo_HG-U133_Plus_2_D02_257672 | KMRC1_KIDNEY | >8 | resistant |
| PARES_p_NCLE_RNA_Redo_HG-U133_Plus_2_C07_257658 | KMRC2_KIDNEY | >8 | resistant |
| BUNDS_p_NCLE_RNA5_HG-U133_Plus_2_G05_419804 | KMS11_HAEMATOPOIETIC_AND_LYMPHOID_TISSUE | >8 | resistant |
| METIS_p_NCLE_RNA1_Human_U133_Plus_2.0_F04_240976 | KMS12BM_HAEMATOPOIETIC_AND_LYMPHOID_TISSUE | >8 | resistant |
| BUNDS_p_NCLE_RNA5_HG-U133_Plus_2_G06_419798 | KP4_PANCREAS | >8 | resistant |
| GILDS_p_NCLE_RNA11_Redo_HG-U133_Plus_2_E11_587582 | KYM1_SOFT_TISSUE | >8 | resistant |
| PARES_p_NCLE_RNA_Redo_HG-U133_Plus_2_E02_257696 | KYSE510_OESOPHAGUS | >8 | resistant |
| METIS_p_NCLE_RNA1_Human_U133_Plus_2.0_G09_241010 | L33_PANCREAS | >8 | resistant |
| WATCH_p_NCLE_RNA8_HG-U133_Plus_2_E07_474598 | L428_HAEMATOPOIETIC_AND_LYMPHOID_TISSUE | >8 | resistant |
| PARES_p_NCLE_RNA_Redo_HG-U133_Plus_2_D08_257684 | LOXIMVI_SKIN | >8 | resistant |
| CRAZY_p_NCLE_RNA10_HG-U133_Plus_2_C01_569564 | LS513_LARGE_INTESTINE | >8 | resistant |
| BUNDS_p_NCLE_RNA5_HG-U133_Plus_2_B12_419712 | LU99_LUNG | >8 | resistant |
| METIS_p_NCLE_RNA1_Human_U133_Plus_2.0_F02_240972 | MALME3M_SKIN | >8 | resistant |
| MAKER_p_NCLE_RNA7_HG-U133_Plus_2_G05_454738 | MCF7_BREAST | >8 | resistant |
| CASED_p_NCLE_RNA4_HG-U133_Plus_2_C09_383596 | MDAMB436_BREAST | >8 | resistant |
| METIS_p_NCLE_RNA1_Human_U133_Plus_2.0_H10_241036 | MDAMB453_BREAST | >8 | resistant |
| PARES_p_NCLE_RNA_Redo_HG-U133_Plus_2_A02_257600 | MDAMB468_BREAST | >8 | resistant |
| NIECE_p_NCLE_RNA3_HG-U133_Plus_2_A06_296000 | MELHO_SKIN | >8 | resistant |
| MAKER_p_NCLE_RNA7_HG-U133_Plus_2_D05_454642 | MG63_BONE | >8 | resistant |
| CASED_p_NCLE_RNA4_HG-U133_Plus_2_H01_383610 | MJ_HAEMATOPOIETIC_AND_LYMPHOID_TISSUE | >8 | resistant |
| SILOS_p_NCLE_RNA9_HG-U133_Plus_2_G03_523458 | MONOMAC1_HAEMATOPOIETIC_AND_LYMPHOID_TISSUE | >8 | resistant |
| MAKER_p_NCLE_RNA7_HG-U133_Plus_2_D03_454638 | MPP89_PLEURA | >8 | resistant |
| PARES_p_NCLE_RNA_Redo_HG-U133_Plus_2_E09_257710 | NCIH1299_LUNG | >8 | resistant |
| AGENT_p_NCLE_RNA6_HG-U133_Plus_2_E04_436560 | NCIH1341_LUNG | >8 | resistant |
| CASED_p_NCLE_RNA4_HG-U133_Plus_2_B05_383744 | NCIH1563_LUNG | >8 | resistant |
| AGENT_p_NCLE_RNA6_HG-U133_Plus_2_G07_436502 | NCIH1666_LUNG | >8 | resistant |
| WATCH_p_NCLE_RNA8_HG-U133_Plus_2_B11_474698 | NCIH1944_LUNG | >8 | resistant |
| METIS_p_NCLE_RNA1_Human_U133_Plus_2.0_H03_241022 | NCIH1975_LUNG | >8 | resistant |
| CASED_p_NCLE_RNA4_HG-U133_Plus_2_F12_383630 | NCIH2052_PLEURA | >8 | resistant |
| CRAZY_p_NCLE_RNA10_HG-U133_Plus_2_G09_569586 | NCIH2172_LUNG | >8 | resistant |
| AGENT_p_NCLE_RNA6_HG-U133_Plus_2_G06_436504 | NCIH226_LUNG | >8 | resistant |
| MAKER_p_NCLE_RNA7_HG-U133_Plus_2_C08_454624 | NCIH2452_PLEURA | >8 | resistant |
| CASED_p_NCLE_RNA4_HG-U133_Plus_2_B06_383736 | NCIH358_LUNG | >8 | resistant |
| BRAKE_p_NCLE_RNA2_HG-U133_Plus_2_H05_241218 | NCIH460_LUNG | >8 | resistant |
| BUNDS_p_NCLE_RNA5_HG-U133_Plus_2_H06_419836 | NUGC4_STOMACH | >8 | resistant |
| AGENT_p_NCLE_RNA6_HG-U133_Plus_2_H02_436488 | OC316_OVARY | >8 | resistant |
| CASED_p_NCLE_RNA4_HG-U133_Plus_2_E07_383688 | OCUM1_STOMACH | >8 | resistant |
| MAKER_p_NCLE_RNA7_HG-U133_Plus_2_H01_454722 | ONS76_CENTRAL_NERVOUS_SYSTEM | >8 | resistant |
| CRAZY_p_NCLE_RNA10_HG-U133_Plus_2_H02_569500.CEL | OVMANA_OVARY | >8 | resistant |
| BRAKE_p_NCLE_RNA2_HG-U133_Plus_2_A09_241058 | OVTOKO_OVARY | >8 | resistant |
| NIECE_p_NCLE_RNA3_HG-U133_Plus_2_F08_296124 | PANC0327_PANCREAS | >8 | resistant |
| WATCH_p_NCLE_RNA8_HG-U133_Plus_2_B06_474580 | PFEIFFER_HAEMATOPOIETIC_AND_LYMPHOID_TISSUE | >8 | resistant |
| GILDS_p_NCLE_RNA11_Redo_HG-U133_Plus_2_G10_587714 | PK45H_PANCREAS | >8 | resistant |
| NIECE_p_NCLE_RNA3_HG-U133_Plus_2_B10_296032 | PK59_PANCREAS | >8 | resistant |
| NIECE_p_NCLE_RNA3_HG-U133_Plus_2_E05_296094 | QGP1_PANCREAS | >8 | resistant |
| BRAKE_p_NCLE_RNA2_HG-U133_Plus_2_C12_241112 | RERFLCAI_LUNG | >8 | resistant |
| MAKER_p_NCLE_RNA7_HG-U133_Plus_2_G02_454712 | RKO_LARGE_INTESTINE | >8 | resistant |
| NIECE_p_NCLE_RNA3_HG-U133_Plus_2_H02_296160 | RT4_URINARY_TRACT | >8 | resistant |
| CASED_p_NCLE_RNA4_HG-U133_Plus_2_E08_383690 | SAOS2_BONE | >8 | resistant |
| CASED_p_NCLE_RNA4_HG-U133_Plus_2_E11_383702 | SCC9_UPPER_AERODIGESTIVE_TRACT | >8 | resistant |
| AGENT_p_NCLE_RNA6_HG-U133_Plus_2_C07_436540 | SF126_CENTRAL_NERVOUS_SYSTEM | >8 | resistant |
| WATCH_p_NCLE_RNA8_HG-U133_Plus_2_B02_474740 | SHP77_LUNG | >8 | resistant |
| WATCH_p_NCLE_RNA8_HG-U133_Plus_2_F05_474682 | SIMA_AUTONOMIC_GANGLIA | >8 | resistant |
| CRAZY_p_NCLE_RNA10_HG-U133_Plus_2_H03_569456 | SKCO1_LARGE_INTESTINE | >8 | resistant |
| CRAZY_p_NCLE_RNA10_HG-U133_Plus_2_G06_569542 | SKHEP1_LIVER | >8 | resistant |
| METIS_p_NCLE_RNA1_Human_U133_Plus_2.0_C11_240918 | SKMEL24_SKIN | >8 | resistant |
| PARES_p_NCLE_RNA_Redo_HG-U133_Plus_2_B06_257632 | SKMEL5_SKIN | >8 | resistant |
| CASED_p_NCLE_RNA4_HG-U133_Plus_2_G03_383634 | SKNAS_AUTONOMIC_GANGLIA | >8 | resistant |
| MAKER_p_NCLE_RNA7_HG-U133_Plus_2_H04_454728 | SKNDZ_AUTONOMIC_GANGLIA | >8 | resistant |
| AGENT_p_NCLE_RNA6_HG-U133_Plus_2_C10_436514 | SNGM_ENDOMETRIUM | >8 | resistant |
| CASED_p_NCLE_RNA4_HG-U133_Plus_2_G05_383642 | SNU398_LIVER | >8 | resistant |
| PARES_p_NCLE_RNA_Redo_HG-U133_Plus_2_D10_257688 | SQ1_LUNG | >8 | resistant |
| CRAZY_p_NCLE_RNA10_HG-U133_Plus_2_G05_569418 | SUPM2_HAEMATOPOIETIC_AND_LYMPHOID_TISSUE | >8 | resistant |
| NIECE_p_NCLE_RNA3_HG-U133_Plus_2_G09_296150 | SW48_LARGE_INTESTINE | >8 | resistant |
| AGENT_p_NCLE_RNA6_HG-U133_Plus_2_E08_436474 | SW480_LARGE_INTESTINE | >8 | resistant |
| METIS_p_NCLE_RNA1_Human_U133_Plus_2.0_G04_241000 | SW620_LARGE_INTESTINE | >8 | resistant |
| METIS_p_NCLE_RNA1_Human_U133_Plus_2.0_D12_240944 | TOV21G_OVARY | >8 | resistant |
| CASED_p_NCLE_RNA4_HG-U133_Plus_2_D10_383716 | U2OS_BONE | >8 | resistant |
| PARES_p_NCLE_RNA_Redo_HG-U133_Plus_2_C03_257650 | U87MG_CENTRAL_NERVOUS_SYSTEM | >8 | resistant |
| PARES_p_NCLE_RNA_Redo_HG-U133_Plus_2_E01_257694 | UACC257_SKIN | >8 | resistant |
| BRAKE_p_NCLE_RNA2_HG-U133_Plus_2_E09_241154 | UACC62_SKIN | >8 | resistant |
| SILOS_p_NCLE_RNA9_HG-U133_Plus_2_C01_523502 | UACC812_BREAST | >8 | resistant |
| AGENT_p_NCLE_RNA6_HG-U133_Plus_2_A07_436590 | UMUC3_URINARY_TRACT | >8 | resistant |
| BUNDS_p_NCLE_RNA5_HG-U133_Plus_2_G12_419818 | VMRCRCW_KIDNEY | >8 | resistant |
| METIS_p_NCLE_RNA1_Human_U133_Plus_2.0_G06_241004 | VMRCRCZ_KIDNEY | >8 | resistant |
| BRAKE_p_NCLE_RNA2_HG-U133_Plus_2_B12_241088 | WM115_SKIN | >8 | resistant |
| CRAZY_p_NCLE_RNA10_HG-U133_Plus_2_D06_569514 | WM1799_SKIN | >8 | resistant |
| PARES_p_NCLE_RNA_Redo_HG-U133_Plus_2_C12_257668 | WM2664_SKIN | >8 | resistant |
| CRAZY_p_NCLE_RNA10_HG-U133_Plus_2_D03_569540 | WM793_SKIN | >8 | resistant |
| CRAZY_p_NCLE_RNA10_HG-U133_Plus_2_D08_569582 | WM88_SKIN | >8 | resistant |
| WATCH_p_NCLE_RNA8_HG-U133_Plus_2_B07_474632 | ZR7530_BREAST | >8 | resistant |
